# Effects of aging on the effectiveness of smoking cessation medication

**DOI:** 10.18632/oncotarget.9090

**Published:** 2016-04-28

**Authors:** Jaqueline Scholz, Paulo Caleb Junior Lima Santos, Carolina Giusti Buzo, Neuza Helena Moreira Lopes, Tania Marie Ogawa Abe, Patricia Viviane Gaya, Humberto Pierri, Clarice Amorim, Alexandre Costa Pereira

**Affiliations:** ^1^ Smoking Cessation Program Department, Heart Institute (InCor), University of São Paulo Medical School, São Paulo, Brazil; ^2^ Laboratory of Genetics and Molecular Cardiology, Heart Institute (InCor), University of São Paulo Medical School, São Paulo, Brazil; ^3^ Cardio Geriatric Clinical Unit, Heart Institute (InCor), University of São Paulo Medical School, São Paulo, Brazil; ^4^ Fleury Group, São Paulo, Brazil; ^5^ Oregon State University, Corvallis, OR, USA

**Keywords:** nicotine replacement therapy, varenicline, bupropion, smoking cessation, aging, Gerotarget

## Abstract

**Background:**

Considering the pharmacokinetic and pharmacodynamic aspects of different medications, it is plausible that the age of a smoker could affect the half-life of these drugs. The aim of this study was to compare the effectiveness of smoking cessation drugs (nicotine replacement therapy, bupropion, and varenicline) used either in isolation or in combination in adults under and over 60 years of age.

**Methods:**

Data were collected from 940 Brazilian patients participating in a smoking cessation program. Participants were prescribed smoking cessation medication to be used for at least 12 weeks and were followed for 52 weeks.

**Results:**

Cessation rates were significantly different among younger and older participants who were using nicotine replacement therapy (NRT) alone. Being over 60 years of age was significantly associated with increased cessation success among those who used NRT alone (OR 2.34, 95% CI: 1.36 to 4.04, *p* = 0.002). The effectiveness of varenicline and bupropion were not significantly different according to age groups.

**Conclusion:**

Using age as a predictor for tailoring smoking cessation drugs might potentially lead to a more individualized prescription of smoking cessation therapy. These results should be tested in randomized controlled trials.

## INTRODUCTION

Smoking is an important risk factor for several chronic conditions, including cardiovascular and lung disease, diabetes, and cancer. Tobacco smoking is and will continue to be a significant public health issue. Currently, over 5 million people die from tobacco-related diseases every year, and the number of deaths is estimated to rise to 8 million by 2030 [1–5].

Smoking cessation has individual and social benefits widely recognized. Large scale and multicenter studies have observed the health benefits of smoking cessation in all age groups: one study demonstrated that younger and older European patients who quit smoking reduced their mortality risk and improved their quality of life [6]. At the individual level, cessation decreases the incidence of chronic diseases as early as the first year of abstinence; cessation benefits also extend to acute health events, and accrue over time [4]. At the social level, decreasing the number of smokers cuts public health expenses by reducing hospitalization rates and the treatment of smoking co-morbidities [7, 8].

Several therapeutic options can assist patients in smoking cessation; these include nicotine replacement therapy (NRT) and drugs like bupropion and varenicline [9]. Considering the pharmacokinetic and pharmacodynamic aspects of these tobacco cessation drugs, it is plausible that the age of the smoker could affect the half-life of these medications therefore altering effectiveness rates. Aging has been shown to affect renal function and also hepatic function [10]. Changes in the glomerular filtration rate due to aging have also been observed by several studies [11, 12]. The mean rate of decline in creatinine clearance was found to be 0.75 mL/min per year, and it was greater in patients with hypertension, which also increases in prevalence as the population ages [11, 12].

Smoking cessation strategies for older adults, particularly, should be individualized and constantly re-evaluated, because this age group has specific metabolic characteristics, comorbidities, and medication use [13]. Furthermore, there are limitations of antismoking drug available in public services and the NRT, widely used, is an over-the-counter drug. In this scenario, this study aimed to compare success rates of different smoking cessation approaches among younger and older patients.

## RESULTS

Of the 940 patients included in this study, 239 (25.4%) were over 60 years, with a mean age of 69 ± 6 years; while 701 patients (74.6%) were 59 years or younger and had a mean age of 48 ± 8 years. Table 1 shows demographic and clinical characteristics of participating patients according to age over 60 years.

**Table 1:** Demographic and clinical characteristics of participating patients according to age over 60 years

	< 60 years *n* = 701	≥ 60 years *n* = 239	*p* value
**Gender, female (%)**	53.4	47.2	0.10
**Ethnicity, White (%)**	65.4	69.8	0.28
**Fagerstrom score**	6.3± 2.8	6.0± 2.6	0.06
**Hypertension (%)**	46.1	65.7	<0.001
**Coronary artery disease (%)**	17.8	30.6	<0.001
**Acute myocardial infarction (%)**	20.6	29.1	<0.01
**Dyslipidemia (%)**	42.9	53.6	<0.01
**Diabetes mellitus type 2 (%)**	11.5	16.6	0.03
**Obesity (%)**	8.1	8.3	0.94
**Depression (%)**	23.8	22.3	0.61
**Anxiety (%)**	19.3	15.5	0.16
**Obstructive pulmonary chronic disease (%)**	14.4	32.1	<0.001
**Asthma (%)**	4.0	2.3	0.18
**Number of diagnosed diseases**	2.4± 1.8	3.2± 1.8	<0.001
**Number of medicaments**	3.2± 3.3	4.3± 3.3	<0.001

Table 2 shows the cessation success rates according to pharmacotherapy and age. Overall, 35.4% of the younger participants achieved cessation, compared to 41% of the older participants. Cessation rates for different age groups were only significantly different among those who were using NRT alone. Older participants who were using NRT alone were significantly more likely to achieve cessation than younger participants using the same medication. Table 3 displays the results of a multivariate analysis of cessation success rates in 302 patients who received NRT alone. Age was the only statistically significant covariate: being over 60 years of age significantly predicted cessation success when NRT alone was used (OR 2.34, 95% CI: 1.36 to 4.04, p = 0.002).

**Table 2:** Smoking cessation success rates according to pharmacotherapy and age

	Success rate (%)		
Pharmacotherapy	< 60 years old	≥ 60 years old	*p*-value
**Overall (*n* = 940)**	35.4	41.0	0.12
**Varenicline (*n* = 246)**	39.8	37.8	0.80
**Varenicline and bupropion (*n* = 108)**	49.4	36.4	0.25
**Bupropion and NRT gum (*n* = 183)**	30.6	34.7	0.60
**Bupropion and NRT patch or/and NRT gum (*n* = 101)**	36.8	48.0	0.32
**NRT alone (*n* = 302)**	27.0	45.9	0.001

NRT: nicotine replacement therapy (patch and/or gum).

Successful smoking cessation was defined as continuous abstinence for 52 weeks.

**Table 3:** Multivariate logistic regression of factors associated with smoking cessation success among patients receiving NRT alone (*n* = 302)

Variables	OR	95% CI	*p* value
**Age over 60 years old**	2.34	1.36-4.04	0.002
**Sex**	0.88	0.50-1.54	0.64
**Race/Ethnicity**	0.82	0.59-1.14	0.24
**Fagerstrom score**	0.93	0.84-1.03	0.17
**Number of diagnosed conditions**	0.97	0.81-1.15	0.71
**Previous cessation attempts**	1.01	0.88-1.16	0.86
**Number of prescribed medications**	0.98	0.89-1.08	0.77

## DISCUSSION

Smoking is an important risk factor for several conditions that are common in older age, such as cardiovascular disease, hypertension, stroke, myocardial infarction, and atherosclerosis [4]. However, smoking cessation is important at all ages. Cessation treatment for older adults requires that health professionals be mindful of patients’ individual needs, degree of dependence, medication use, and co-morbidities [9].

Current research indicates that pharmacotherapy increases cessation success [14–17]. Previous studies, however, have indicated that NRT alone was less effective than bupropion and varenicline; and that it should be used in addition to these medications [18–20]. This study, on the other hand, found that NRT alone was significantly more effective at promoting cessation for older group, compared with younger group. The success rates for older group using NRT alone were equal or better than those of older group using bupropion and varenicline. NRT might be an important drug in smoking cessation especially in elderly patients, based on our results.

The pathway that explains the effectiveness of NRT for older group is still not fully understood. A pharmacokinetic trial demonstrated that the disposition of bupropion was similar in younger and older participants [21]. Regarding the pharmacokinetics of varenicline, data were pooled from 9 clinical studies that included a total of 1878 subjects; after accounting for patients’ renal function, researchers found no apparent effect of age, sex, or race on varenicline's pharmacokinetics [22]. On the other hand, previous research indicated that nicotine, like many other chemical and biological compounds, may be metabolized differently depending on sex and race/ethnicity [23–25]. However, studies analyzing the impact of age differences in the metabolism of nicotine were conducted with rats and they found that younger rats metabolized nicotine faster than older rats did [26, 27]. Similar metabolic reactions in humans could explain why we observed a different effectiveness of NRT for older group.

This study has some limitations that should be considered. First of all, patients were not randomly assigned smoking cessations drugs, but rather were intentionally prescribed the drugs that were best suited for their individual circumstances. In addition, data were collected from patients who voluntarily sought and joined a smoking cessation program, which indicates a certain degree of willingness to quit smoking; thus, the sample may not be representative of the regular population. In addition, the patients themselves administered cessation treatments, making it hard to assess compliance over time. Further studies (ideally clinical trials) are needed to validate these results in a larger and more representative sample; researchers should also focus on gaining a better understanding of the biochemical mechanisms that explain successful cessation and how they may differ between younger and older groups.

Nevertheless, these results are noteworthy for clinical practice and public health efforts, because they indicate that NRT alone can be an effective and safe option for promoting tobacco cessation among older adults [14, 28, 29]. Also, a recent randomized trial proved that treating slow metabolizers with the nicotine patch could optimize quit rates while minimizing side effects [30]. Among 1246 participants (662 slow metabolizers of nicotine, 584 normal metabolizers of nicotine) were enrolled and randomly assigned to 3 interventions (408 placebo, 418 nicotine patch, 420 varenicline).

In conclusion, using age as a predictor for tailoring smoking cessation drugs might potentially lead to a more individualized prescription of smoking cessation therapy. These results should be tested in randomized controlled trials.

## MATERIAL AND METHODS

### Study Design

This was an outcome research that utilized a retrospective cohort study design from PAF (Programa de Assistência ao Fumante / Program of Assistance to Smokers). The study protocol was approved by the Ethics Committee of the Heart Institute (InCor), University of São Paulo Medical School (0022/11). Eligible patients who wished to begin smoking cessation treatment were admitted to the smoking cessation program, from January 2008 to December 2011, in the InCor, a tertiary hospital. Of the 1053 patients who consented to having their information analyzed, 940 completed follow-up and 113 dropped out of the study because they did not start the drug treatment. Patients received individual medical advice and cessation drugs to be used for at least 12 weeks. The available medications included NRT (patch and gum), bupropion, and varenicline. These drugs were initiated as monotherapy according to the nicotine dependence level of the patient, previous use of smoking cessation medication, availability of medication, and contraindications. NRT was prescribed preferentially to patients from SUS (Brazilian system of health) and who smoked at least 20 cigarettes per day. Bupropion was prescribed to SUS patients who smoked more than 20 cigarettes per day or women who smoked at least 20 cigarettes per day. These drugs could be combined to help a patient achieve the smoke-free status or decrease withdrawal symptoms. For this study, varenicline was available for use by SUS patients. In this case, varenicline was used in patients who failed to stop smoking in previous attempts with NRT and/or bupropion, or who smoked one or more pack(s) of cigarettes per day. Our indication to start providing bupropion at 150 mg/day was if the patient did not achieve complete abstinence 2 or 3 weeks after starting varenicline, or if the patient achieved complete abstinence, but experienced moderate or intense withdrawal symptoms.

At the end of the study, patients were divided into 3 groups according to their smoking status: success (continuous abstinence for 52 weeks), relapse (abstinence interrupted prior to completing the 52 weeks), and failure (no abstinence over the first 8 weeks of treatment). We then analyzed the effectiveness of NRT, bupropion, and varenicline (alone or in combination) in patients under 60 years of age and over 60 years of age.

### Statistical analysis

Categorical variables are presented as percentages, and continuous variables are presented as means ± SD (standard deviation). Chi-square and Student t-tests were performed to analyze demographic and clinical data, including success rates, according to age, sex, and prescribed pharmacotherapy. Univariate and multivariate logistic regression were used to estimate the odds ratio (OR) of cessation success in patients using NRT. Older patient was classified using a cut-off of 60 years according to the WHO for developing countries. Models were developed using the following variables: age, sex, race, Fagerstrom score, number of co-morbidities, previous cessation attempts, and number of prescribed medications. Statistical analyses were carried out using SPSS 16.0 software (IBM, New York, NY), with the level of significance set at p ≤ 0.05.

## References

[1] Beaglehole R, Bonita R, Horton R, Adams C, Alleyne G, Asaria P, Baugh V, Bekedam H, Billo N, Casswell S, Cecchini M, Colagiuri R, Colagiuri S (2011). Priority actions for the non-commuunicable disease crisis. Lancet.

[2] Calhoun DA, Jones D, Textor S, Goff DC, Murphy TP, Toto RD, White A, Cushman WC, White W, Sica D, Ferdinand K, Giles TD, Falkner B (2008). Resistant hypertension: diagnosis, evaluation, and treatment. Hypertension.

[3] Glantz S, Gonzalez M (2012). Effective tobacco control is key to rapid progress in reduction of non-communicable diseases. Lancet.

[4] U.S. Department of Health and Human Services The Health Consequences of Smoking—50 Years of Progress: A Report of the Surgeon General.

[5] World Health Organization (2009). Report on the global tobacco epidemic 2009: implementing smoke-free environments.

[6] Haveman-Nies A, de Groot LCPGM, van Staveren WA (2003). Dietary quality, lifestyle factors and healthy ageing in Europe: the SENECA study. Age Aging.

[7] Fichtenberg CM, Glantz SA (2000). Association of the California Tobacco Control Program with declines in cigarette consumption and mortality from heart disease. N Engl J Med.

[8] Lightwo8od JM, Glantz SA (1997). Short-term economic and health benefits of smoking cessation: myocardial infarction and stroke. Circulation.

[9] US Public Health Service Report (2008). A Clinical Practice Guideline for Treating Tobacco Use and Dependence: 2008 Update. Am J Prev Med.

[10] Turnheim K (2003). When drug therapy gets old: pharmacokinetics and pharmacodynamics in the elderly. Exp Gerontol.

[11] Lindeman RD, Tobin JD, Shock NW (1984). Association between blood pressure and the rate of decline in renal function with age. Kidney Int.

[12] Rodríguez-Puyol D (1998). The aging kidney. Kidney Int.

[13] Appel DW, Aldrich TK (2003). Smoking cessation in the elderly. Clin Geriatr Med.

[14] Stead LF, Perera R, Bullen C, Mant D, Hartmann-Boyce J, Cahill K, Lancaster T (2012). Nicotine replacement therapy for smoking cessation. Cochrane Database of Systematic Reviews.

[15] Dale LC, Glover ED, Sachs DL, Schroeder DR, Offord KP, Croghan IT, Hurt RD (2001). Bupropion For Smoking Cessation: Predictors Of Successful Outcome. Chest.

[16] Jorenby DE, Leischow SJ, Nides MA, Rennard SI, Johnston JA, Hughes AR, Smith SS, Muramoto ML, Daughton DM, Doan K, Fiore MC, Baker TB (1999). A controlled trial of sustained-release bupropion, a nicotine patch, or both for smoking cessation. N Engl J Med.

[17] Scholz J, Portela LD, Abe TO, Gaya PV, Santos VG, Ferreira C, Amorin C, Pereira AC, Santos PCJL (2016). Cost-effectiveness analysis of smoking-cessation treatment using electronic medical records in a cardiovascular hospital. Clinical Trials and Regulatory Science in Cardiology.

[18] Issa JS, Abe TO, Moura S, Santos PC, Pereira AC (2013). Effectiveness of coadministration of varenicline, bupropion, and serotonin reuptake inhibitors in a smoking cessation program in the real-life setting. Nicotine Tob Res.

[19] Jorenby DE, Hays JT, Rigotti NA, Azoulay S, Watsky EJ, Williams KE, Billing CB, Gong J, Reeves KR, Varenicline Phase 3 Study Group (2006). Efficacy of varenicline, an alpha4beta2 nicotinic acetylcholine receptor partial agonist, vs placebo or sustained-release bupropion for smoking cessation: a randomized controlled trial. JAMA.

[20] Aubin H-J, Bobak A, Britton JR, Oncken C, Billing CB, Gong J, Williams KE, Reeves KR (2008). Varenicline versus transdermal nicotine patch for smoking cessation: results from a randomised open-label trial. Thorax.

[21] Preskorn SH, Othmer SC (1984). Evaluation of bupropion hydrochloride: the first of a new class of atypical antidepressants. Pharmacotherapy.

[22] Ravva Pl, Gastonguay MR, Tensfeldt TG, Faessel HM (2009). Population pharmacokinetic analysis of varenicline in adult smokers. JClinPharmacol.

[23] Benowitz NL (1999). Nicotine addiction. Prim Care.

[24] Benowitz NL, Perez-Stable EJ, Herrera B, Jacob P (2002). Slower metabolism and reduced intake of nicotine from cigarette smoking in Chinese-Americans. J Natl Cancer Inst.

[25] Berg JZ, Mason J, Boettcher AJ, Hatsukami DK, Murphy SE (2010). Nicotine metabolism in African Americans and European Americans: variation in glucuronidation by ethnicity and UGT2B10 haplotype. J Pharmacol Exp Ther.

[26] Vieira-Brock PL, Andrenyak DM, Nielsen SM, Fleckenstein AE, Wilkins DG (2013). Age-related differences in the disposition of nicotine and metabolites in rat brain and plasma. Nicotine Tob Res.

[27] Craig EL, Zhao B, Cui JZ, Novalen M, Miksys S, Tyndale RF (2014). Nicotine pharmacokinetics in rats is altered as a function of age, impacting the interpretation of animal model data. Drug Metab Dispos.

[28] Benowitz NL, Perez-Stable EJ, Fong I, Modin G, Herrera B, Jacob P (1999). Ethnic differences in N-glucuronidation of nicotine and cotinine. J Pharmacol Exp Ther.

[29] Jarvis MJ (2014). Why people smoke. BMJ.

[30] Lerman C, Schnoll RA, Hawk LW, Cinciripini P, George TP, Wileyto EP, Swan GE, Benowitz NL, Heitjan DF, Tyndale RF, PGRN-PNAT Research Group (2015). Use of nicotine metabolite ratio as a genetically informed biomarker of response to nicotine patch or varencline for smoking cessation: a randomised, double-blind placebo-controlled trial. Lancet Resp Med.

